# Long-term impacts of COVID-19 in patients with prior heart failure in Korea: A nationwide cohort study using the common data model

**DOI:** 10.1097/MD.0000000000039236

**Published:** 2024-08-02

**Authors:** Seunghwa Lee, Kyoung Ree Lim, Kwang Jin Chun, Bum Sung Kim

**Affiliations:** a Division of Cardiology, Department of Medicine, Wiltse Memorial Hospital, Suwon, Gyeonggi-do, South Korea; b Division of Infectious Diseases, Department of Internal Medicine, Kyung Hee University Hospital at Gangdong, Seoul, South Korea; c Division of Cardiology, Department of Internal Medicine, Kangwon National University Hospital, Kangwon National University School of Medicine, Chuncheon, South Korea; d Division of Cardiology, Department of Medicine, Konkuk University Medical Center, Seoul, South Korea.

**Keywords:** COVID-19, heart failure, mortality, postacute sequelae

## Abstract

Limited data are available on the long-term prognosis and monitoring period after coronavirus disease 2019 (COVID-19) infection in the population with prior heart failure (HF). We aimed to exam the association of COVID-19 with clinical prognosis in populations with prior HF and evaluate prognosis within 30 days and 30 days to 1 year after infection. Based on insurance benefit claims sent to the Health Insurance Review and Assessment Service of Korea from January 2018 to April 2022, 9,822,577 patients were selected and converted to the Observational Medical Outcomes Partnership-common data model by the Big Data Department of Health Insurance Review and Assessment Service of Korea. In the dataset, 1,565,274 patients exhibited diagnosis of HF based on the International Statistical Classification of Diseases and Related Health Problems 10 codes. They were divided into 2 groups according to COVID-19 infection, and propensity-score-matching analysis was performed. The clinical outcome was all-cause mortality. Among the 1,565,274 patients with an HF diagnosis, 1,152,975 patients were classified into the HF with the COVID-19 group and 412,299 patients in the HF without COVID-19 group. We created 200,780 matched pairs by propensity-score-matching analysis. Within 30 days of COVID-19, the HF with COVID-19 group had a higher risk of all-cause death compared with the HF without COVID-19 group (hazard ratio [HR]: 2.19, 95% confidence interval [CI]: 2.04–2.36, *P *< .01). Thirty days to 1 year after COVID-19 infection, the HF with COVID-19 group exhibited a higher risk of all-cause death (HR: 2.04, 95% CI: 1.83–2.27, *P *< .01). In populations with prior HF, COVID-19 is associated with a higher risk of all-cause mortality within 30 days and this risk remains augmented up to 1 year after the acute phase of COVID-19. Our findings suggest that greater attention may be crucial in populations with prior HF for a prolonged period after COVID-19 infection.

## 1. Introduction

The coronavirus disease 2019 (COVID-19) pandemic, caused by severe acute respiratory syndrome coronavirus 2 (SARS-CoV-2), led to various complications of the cardiovascular and respiratory systems and was responsible for unprecedented morbidity, as well as a substantial shift in hospital admissions.^[1–4]^ Several years after the COVID-19 pandemic, SARS-CoV-2 remains present and is transitioning to an endemic status. As the virus has evolved, it continues to pose a health threat in terms of both acute infection and postacute sequelae.^[5]^ In COVID-19 patients, cardiovascular disease is a common comorbidity and has been associated with poor outcomes.^[6]^ Heart failure (HF) is a complex syndrome that is among the leading cardiovascular causes of morbidity and mortality worldwide, and previous research suggests that patients with prior HF were at significant risk for acute decompensation after COVID-19, which was associated with a very high mortality rate.^[7,8]^ Recent studies have demonstrated a significant risk and burden of cardiovascular disease in survivors of acute COVID-19, with some individuals who have recovered from COVID-19 being at an increased risk of developing HF.^[9–11]^ Given the significant impact of HF on morbidity and mortality, it is critical to monitor and manage individuals who have recovered from COVID-19 for potential cardiac manifestations, especially those who have had prior heart failure. However, there is limited data on the long-term prognosis and monitoring period after COVID-19 in the population with prior heart failure. Therefore, we aimed to examine the association of COVID-19 with clinical prognosis in a population with prior HF and evaluate prognosis within 30 days of infection and 30 days to 1 year after infection using deidentified nationwide COVID-19 data from the Republic of Korea.

## 2. Material and methods

### 2.1. Data curation

The current dataset, based on insurance benefit claims sent to the Health Insurance Review and Assessment Service of Korea (HIRA), is composed of all the patients who used National Health Insurance of Korea from January 2018 to April 2022. Among those people, 9,822,577 patients were selected and converted to the Observational Medical Outcomes Partnership (OMOP)-common data model (CDM) by the Big Data Department of HIRA. The name of the database is HIRA_CMD, and the used platform is Oracle. We used the database shared in the form of OMOP-CDM, which has been established as a multi-stakeholder, interdisciplinary collaborative to create open-source solutions that bring out the value of observational health data through large-scale analytics.^[12]^ This was a retrospective observational cohort study conducted in accordance with the principles of the Declaration of Helsinki. The Institutional Review Board of Kon-Kuk University Medical Center waived the need for informed consent for this study (KUMC 2022-08-008) since we used de-identified data based on insurance benefit claims sent to the HIRA. Informed consent was waived due to the retrospective nature of the study.

### 2.2. Cohort definition and outcomes

In the dataset, patients were entered into the cohort if they were 20 years of age or older and had a diagnosis of HF based on appropriate International Statistical Classification of Diseases and Related Health Problems 10 (ICD-10) codes [I11.0 Hypertensive heart disease with (congestive) heart failure; I25.5 Ischemic cardiomyopathy; I42.0 Dilated cardiomyopathy; I42.9 Cardiomyopathy, unspecified; I50.0 Congestive heart failure; I50.1 Left ventricular failure; I50.9 Heart failure, unspecified]. The patients with a diagnosis of HF were divided into 2 groups. The target group included HF patients with COVID-19 infection, while the comparator group was composed of HF patients without COVID-19 infection. Baseline characteristics were retrieved from Observational Health Data Sciences and Informatics (OHDSI) CDM. The clinical outcome was all-cause mortality. For analysis, we investigated the all-cause mortality rate of COVID-19 within 30 days and 30 days to 1 year after COVID-19 infection in patients with the diagnosis of HF. Mortality and time of death were provided by HIRA and have been validated.

### 2.3. Statistical analysis

Analysis tools of the OMOP-CDM are built into the interactive analysis platform ATLAS and the OHDSI Methods Library R packages version 3.5.1 (R Foundation for Statistical Computing, Vienna, Austria). OHDSI’s open-source software (ATLAS version 2.7.6) is publicly available on the GitHub repository (https://github.com/OHDSI/ accessed on December 20, 2022). In addition, concept sets which we used to define baseline characteristics and study outcomes are also available (https://github.com/OHDSI/COVID-19/ accessed on December 20, 2022). A Cox regression analysis was used to evaluate all-cause mortality according to the COVID-19 infection. To retain a large sample size and maximize the study power while maintaining a balance in covariates between the 2 groups, we conducted rigorous adjustments for differences in baseline and characteristics of patients using the weighted Cox proportional-hazards regression models with propensity-score matching.^[13]^ Kaplan–Meier estimates were used to construct survival curves and compare them with the log-rank test. Cox regression was used to evaluate all-cause deaths. All tests were 2-tailed and *P* < .05 was considered statistically significant.

## 3. Results

### 3.1. Baseline characteristics

**Figure 1.**
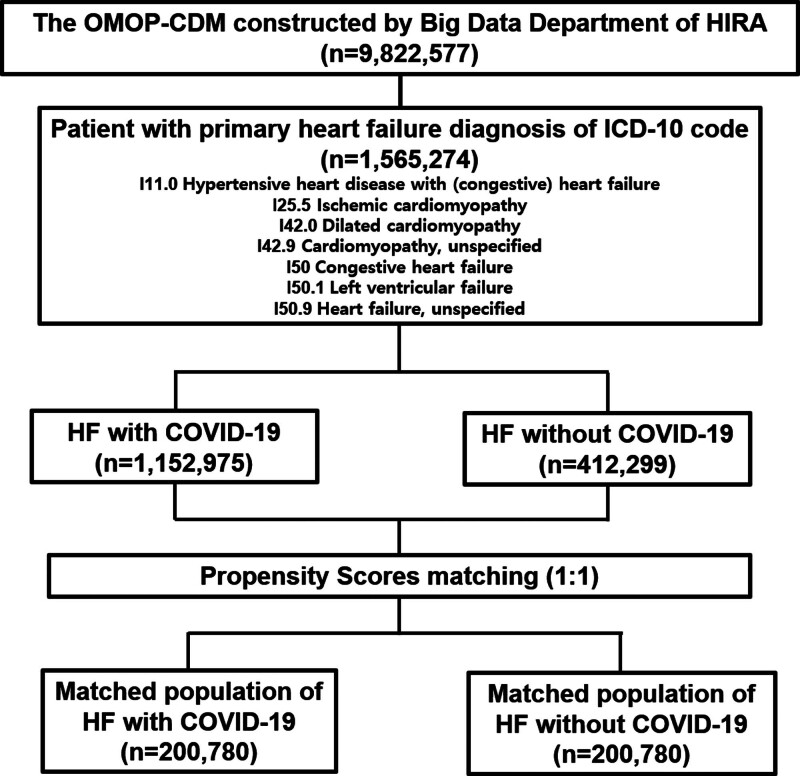
Schema of the study population distribution in the cohort. CDM = common data model, HF = heart failure, HIRA = Health Insurance Review and Assessment Service of Korea, ICD-10 = International Statistical Classification of Diseases and Related Health Problems 10, OMOP = Observational Medical Outcomes Partnership.

**Figure 2.**
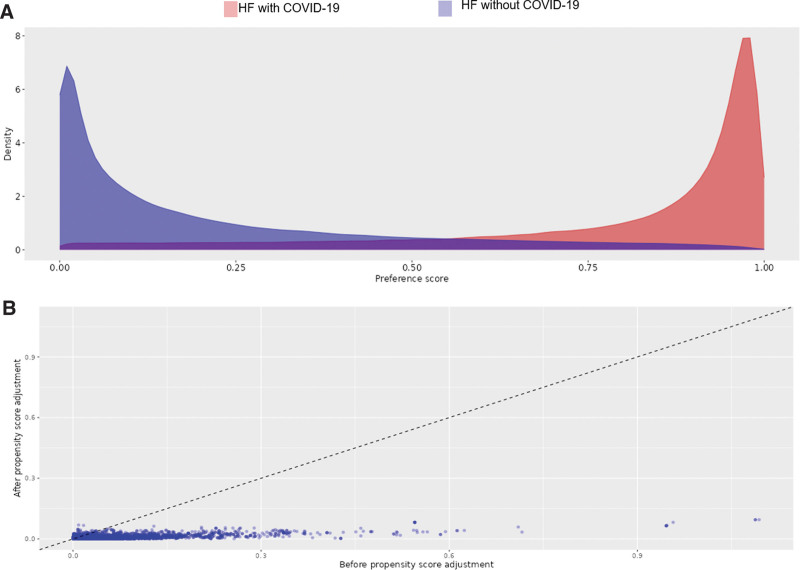
(A) Propensity score, (B) covariate balance of the analysis of all-cause mortality.

Among 9,822,577 patients converted to OMOP-CDM by the Big Data Department of HIRA, a total of 1,565,274 patients were diagnosed with HF and enrolled in the analysis. Among the cohort, 1,152,975 patients had a diagnosis of HF with COVID-19 and were classified into the HF with COVID-19 group, while the remaining 412,299 patients had a diagnosis of HF without COVID-19 and were classified into the HF without COVID-19 group (Fig. 1). We created 200,780 matched and well-balanced pairs of patients by performing propensity-score-matching for the entire cohort (Fig. 2). Table 1 shows the baseline characteristics of the entire cohort population with HF diagnosis according to COVID-19 and we observed no significant imbalances in the baseline variables of the propensity-score-matched population between the 2 groups.

**Table 1:** Baseline characteristics of the cohort population with heart failure according to COVID-19 before and after propensity-score matching.

	Overall population			Propensity-score-matched population		
	HF with COVID-19	HF without COVID-19	SMD	HF with COVID-19	HF without COVID-19	SMD
	(N = 1,152,975)	(N = 412,299)		(N = 200,780)	(N = 200,780)	
Age groups						
20 to 24	7.3	0.9	0.33	1.1	1.5	−0.04
25 to 29	9.1	1.1	0.37	1.4	1.8	−0.04
30 to 34	8.8	1.3	0.35	1.6	2.1	−0.04
35 to 39	9.7	2	0.33	2.5	3.1	−0.04
40 to 44	11.7	2.9	0.34	3.7	4.5	−0.04
45 to 49	9.6	4.6	0.20	5.4	6.3	−0.04
50 to 54	9.2	6.6	0.10	8.1	8.3	−0.01
55 to 59	7.6	9.3	−0.06	10.3	10.2	0.00
60 to 64	9.0	11.8	−0.09	14.0	13.1	0.03
65 to 69	6.9	11.9	−0.17	13.8	12.4	0.04
70 to 74	4.4	13.3	−0.32	12.3	11.6	0.02
75 to 79	2.8	14.2	−0.42	10.2	10.1	0.00
80 to 84	2.1	11.6	−0.38	8.3	8.2	0.01
85 to 89	1.2	6.2	−0.27	4.8	4.6	0.01
90 to 94	0.5	2.0	−0.13	2.0	1.8	0.02
95 to 99	0.1	0.4	−0.05	0.5	0.4	0.01
Gender: female	57.9	51.5	0.13	52.5	25.1	0.00
Medical history: general						
Acute respiratory disease	82.7	50.9	0.72	60.1	58.4	0.03
Chronic liver disease	2.5	7.3	−0.22	6.2	5.7	0.02
Chronic obstructive lung disease	0.9	6.3	−0.30	3.4	3.5	0.00
Crohn disease	0.1	0.1	0.01	0.1	0.1	0.00
Dementia	2.9	8.9	−0.26	9.9	8.3	0.05
Depressive disorder	7.5	16.8	−0.29	15.0	14.0	0.03
Diabetes mellitus	12.4	34.7	−0.54	30.6	28.6	0.04
Gastroesophageal reflux disease	31.3	34.3	−0.06	34.8	32.9	0.04
Gastrointestinal haemorrhage	1.9	3.8	−0.11	3.1	3.0	0.01
Hyperlipidemia	27.6	70.4	−0.95	62.5	59.3	0.06
Hypertensive disorder	22.1	70.0	−1.09	63.7	59.1	0.10
Lesion of liver	1.8	5.0	−0.18	4.2	3.8	0.02
Obesity	0.2	0.2	0.01	0.2	0.2	0.00
Osteoarthritis	13.5	22.6	−0.24	21.9	20.4	0.04
Pneumonia	3.0	8.4	−0.24	6.1	5.9	0.01
Psoriasis	0.8	1.0	−0.03	1.0	0.9	0.00
Rheumatoid arthritis	1.3	2.8	−0.10	2.4	2.2	0.01
Schizophrenia	0.7	1.1	−0.04	1.3	1.1	0.01
Ulcerative colitis	0.1	0.1	0.00	0.1	0.1	0.00
Urinary tract infectious disease	4.0	7.4	−0.14	6.4	5.8	0.02
Visual system disorder	35.7	38.3	−0.05	39.7	37.4	0.05
Medical history: cardiovascular disease						
Cerebrovascular disease	2.8	10.5	−0.32	8.5	7.8	0.03
Peripheral vascular disease	7.5	20.5	−0.38	17.7	16.8	0.03
Pulmonary embolism	0.2	1.8	−0.17	0.8	0.9	−0.01
Venous thrombosis	0.7	2.1	−0.12	1.6	1.5	0.01
Medical history: hematologic neoplasm						
Hematologic neoplasm	0.3	0.6	−0.04	0.6	0.5	0.00
Malignant lymphoma	0.1	0.2	−0.02	0.2	0.2	0.01
Malignant neoplasm of anorectum	0.2	0.4	−0.05	0.3	0.3	0.00
Malignant neoplastic disease	4.3	8.5	−0.17	7.9	7.2	0.03
Malignant tumor of breast	0.6	0.7	−0.01	0.9	0.8	0.01
Malignant tumor of colon	0.3	0.9	−0.09	0.7	0.7	0.00
Malignant tumor of lung	0.1	0.5	−0.06	0.4	0.3	0.01
Malignant tumor of urinary bladder	0.1	0.4	−0.05	0.3	0.3	0.00
Malignant neoplasm of prostate	0.3	1.3	−0.10	1.0	0.9	0.01

Data are presented as percentages.

HF = heart failure, SMD = standardized mean difference.

### 3.2. Clinical outcome

**Figure 3.**
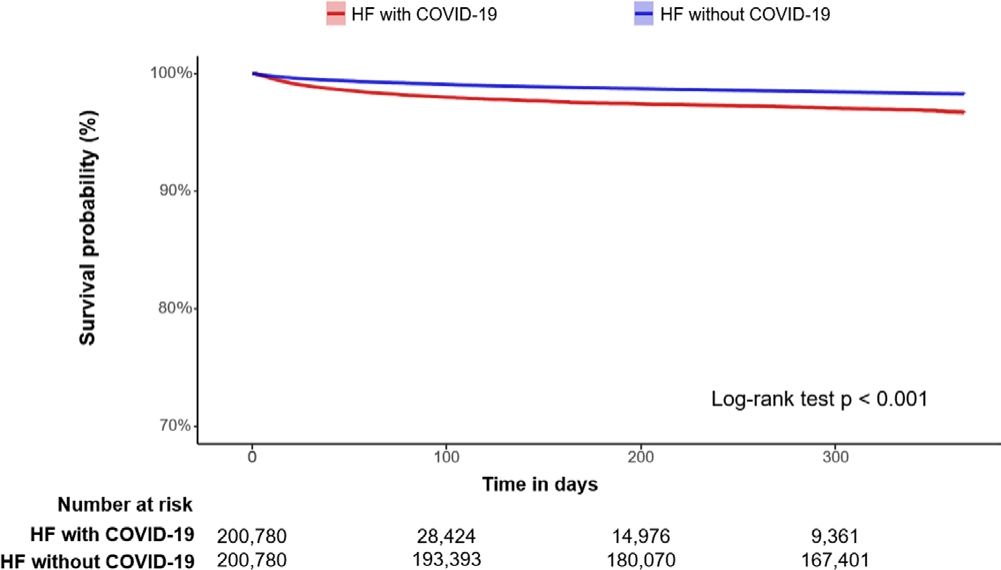
Kaplan–Meier curve of all-cause mortality between heart failure with COVID-19 and heart failure without COVID-19 in the propensity-score-matched population during follow-up. COVID-19 = coronavirus disease 2019, HF = heart failure.

**Figure 4.**
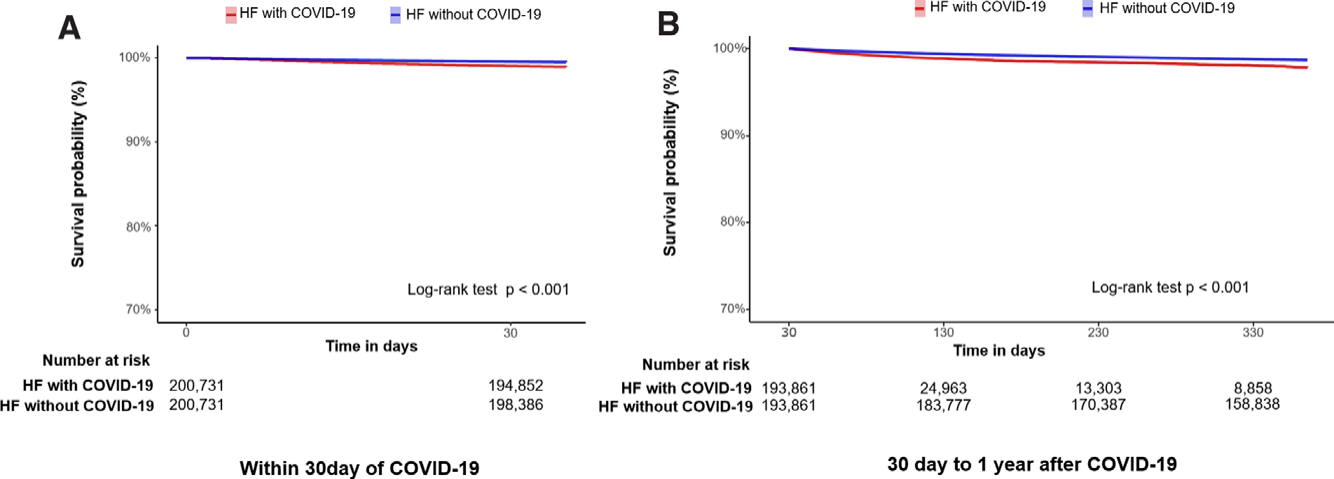
Kaplan–Meier curve of all-cause mortality within 30 d (A) and 30 d to 1 yr after infection (B) between heart failure with COVID-19 and heart failure without COVID-19 in the propensity-score-matched population. COVID-19 = coronavirus disease 2019, HF = heart failure.

Table 2 demonstrates the all-cause mortality of the overall and propensity-score-matched population between the HF with COVID-19 and HF without COVID-19 groups during the 1 year follow-up. In the overall population, the total person-years was 201,827 years in the HF with COVID-19 group and 377,791 years in the HF without COVID-19 group. The incidence of all-cause mortality was 22.16 per 1000 person-years in the HF with COVID-19 group and 26.19 per 1000 person-years in the HF without COVID-19 group. In the propensity-score-matched population, the total person-years was 41,890 years in the HF with COVID-19 group and 182,351 years in the HF without COVID-19 group. The incidence of all-cause mortality was 77.89 per 1000 person-years in the HF with COVID-19 group and 17.92 per 1000 person-years in the HF without COVID-19 group. There was a higher risk of all-cause death in the HF without COVID-19 group (hazard ratio [HR]: 2.24, 95% confidence interval [CI]: 2.11–2.39, *P *< .01) compared to the HF without COVID-19 group. Figure 3 demonstrates the Kaplan–Meier curve depicting hazard for all-cause mortality between the 2 propensity-score-matched groups during the 1-year follow-up period. Within 30 days of COVID-19 infection, compared with the HF without COVID-19 group, the HF with COVID-19 group demonstrated a higher risk of all-cause mortality (HF with COVID-19 group versus HF without COVID-19 group: 131.11 versus 56.69 per 1000 person-years, HR: 2.19, 95% CI: 2.04–2.36, *P *< .01) in the propensity-score-matched population (Table 3). Thirty days to 1 year after COVID-19 infection, compared with the HF without COVID-19 group, the HF with COVID-19 group demonstrated a higher risk of all-cause mortality (39.80 versus 14.07 per 1000 person-years, HR: 2.04, 95% CI: 1.83–2.27, *P *< .01) in the propensity-score-matched population (Table 3). Figure 4 demonstrates the Kaplan–Meier curve depicting hazard for all-cause mortality within 30 days and 30 days to 1 year after COVID-19 infection between the 2 propensity-score-matched groups.

**Table 2:** All-cause mortality between the heart failure with COVID-19 and heart failure without COVID-19 groups in the overall and propensity-score-matched populations.

	Overall population				Propensity-score-matched population			
	HF with COVID-19	HF without COVID-19	HR	*P* value	HF with COVID-19	HF without COVID-19	HR	*P* value
	(N = 1,152,975)	(N = 412,299)	(95% CI)		(N = 200,780)	(N = 200,780)	(95% CI)	
All-cause mortality during the 1 yr follow-up	22.16	26.19	0.39	<.01	77.89	17.92	2.24	<.01
			(0.38–0.41)				(2.11–2.39)	

Data are presented as rate (per 1000 person-years) and HR [95% CI].

Abbreviations: CI = confidence interval, HF = heart failure, HR = hazard ratio.

**Table 3:** All-cause mortality within 30 d and 30 d to 1 yr after infection between the heart failure with COVID-19 and heart failure without COVID-19 groups in the overall and propensity-score-matched populations.

	Overall population				Propensity-score-matched population			
	HF with COVID-19	HF without COVID-19	HR	*P* value	HF with COVID-19	HF without COVID-19	HR	*P* value
	(N = 1,152,975)	(N = 412,299)	(95% CI)		(N = 200,780)	(N = 200,780)	(95% CI)	
All-cause mortality within 30 d	31.17	84.39	0.37	<.01	131.11	56.69	2.19	<.01
			(0.35–0.39)				(2.04–2.36)	
	HF with COVID-19	HF without COVID-19	HR	*P* value	HF with COVID-19	HF without COVID-19	HR	*P* value
	(N = 1,082,229)	(N = 409,060)	(95% CI)		(N = 193,861)	(N = 193,861)	(95% CI)	
All-cause mortality at 30 d to 1 yr	13.19	20.10	0.44	<.01	39.80	14.07	2.04	<.01
			(0.41–0.47)				(1.83–2.27)	

Data are presented as rate (per 1000 person-years) and HR [95% CI].

CI = confidence interval, HF = heart failure, HR = hazard ratio.

## 4. Discussion

In this large-scaled population-based cohort, we observed that the population of prior HF history with concomitant COVID-19 infection had a higher risk of all-cause mortality compared to the matched HF population without COVID-19. This higher risk of all-cause mortality was observed not only within 30 days, but also 30 days to 1 year after COVID-19 infection in the population with prior HF history.

Patients with HF have limited cardiac, renal, and pulmonary reserves, making them more susceptible to insults from infections. Previous knowledge of other respiratory tract infections, such as influenza, have proven the virus potential to trigger the decompensation of HF and have an association with increased in-hospital morbidity and mortality in patients with HF.^[14]^ In the COVID-19 pandemic, infection caused respiratory and cardiovascular complications, which are associated with increasing short-term mortality rates in patients with prior HF history compared to patients without HF history.^[7,8,15]^ In data from the cohort of the general population, exposure to COVID-19 was associated with a risk of adverse cardiovascular events, including HF, especially in patients requiring hospitalization and in the early (first 30 days) postperiod. Moreover, worse cardiovascular outcomes were not confined only within the acute illness phase, but were observed even after 30 days.^[16]^ The previous report by Xie et al^[11]^ provided substantial evidence from the US Department of Veterans Affairs National Healthcare Databases that survivors of acute COVID-19 exhibited increased risk and 12-month burdens of incident cardiovascular disease beyond the first 30 days of infection.

In this study, we used a cohort of a prior HF population from a large COVID-19 national database and analyzed the risk of all-cause mortality within 30 days and 30 days to 1 year later. The risk of all-cause mortality is more than doubled in the acute phase (within 30 days) and elevated risk extends beyond the acute phase (30 days to 1 year later), compared to the prior HF population without COVID-19. Although the COVID-19 pandemic has regressed, SARS-CoV-2 is transitioning to endemic status and infections continue. The sequelae of SARS-CoV-2 infections can involve multiple organ systems and transition to long COVID.^[17]^ The prolonged augmentation of risk of all-cause mortality is a critical issue to monitoring and managing individuals who have recovered from COVID-19, especially those who have a history of heart failure. The finding of this study suggests that COVID-19 infection may be a factor influencing long-term prognosis in populations with prior HF. Therefore, greater attention to cardiovascular risk management in patients with prior HF and a low threshold for cardiovascular investigations of patients exposed to COVID-19 are important for prevention and timely treatment. The underlined mechanisms between COVID-19 and development of cardiovascular disease, especially heart failure development, in the post phase of COVID-19 are not entirely clear.^[17,18]^ Presumed mechanisms include prolonged damage form direct viral invasion of cardiomyocytes and subsequent cell death, endothelial cell infection and transcriptional alteration of multiple cell types in heart tissue, complement activation and complement-mediated coagulopathy and microangiopathy, downregulation of ACE2 and dysregulation of the renin–angiotensin–aldosterone system, autonomic dysfunction.^[19–22]^ Furthermore, SARS-CoV-2 infection can trigger a cytokine storm; many pro-inflammatory cytokines and chemokines, such as TNF-a, IL-1b, and IL-6, are overproduced, damaging various organs, including those in the cardiovascular system and induce subsequent fibrosis and scarring of cardiac tissue.^[19,23]^

The results of this study should be interpreted considering the following limitations. First, our analysis does not consider other potential modifying factors such as the impact of vaccinations, multiple infections of SARS-CoV-2 subtype. In addition, we could not analyze the potential benefits of antiviral treatments like Paxlovid on COVID-19 patients with HF, because CDM does not provide information about the antiviral treatments or vaccinations. Second, this is a retrospective study and thus associated with the inherent limitations of this type of analysis. Third, owing the nature of the database that was retrieved from insurance-issued claims, clinical presentations, symptoms, and hospital courses could not be evaluated. Fourth, the results of the current study were derived from a cohort in the Republic of Korea. Therefore, the impact of ethnicity cannot be analyzed and requires further evaluation. Despite these limitations, this study provides real-world evidence about the long-term prognosis of the HF population after COVID-19 infection and valuable information in the current long COVID-19 situation.

## 5. Conclusion

In populations with prior HF, COVID-19 infection is associated with a higher risk of all-cause mortality within 30 days and these risks remain augmented up to 1 year after the acute phase of COVID-19 infection. Our findings suggest that greater attention may be crucial in the population with prior HF for a prolonged period after COVID-19 infection.

## Acknowledgments

The authors appreciate the Ministry of Health and Welfare and the Health Insurance Review and Assessment Service of Korea for promptly sharing valuable National Health Insurance claims data, and Jinseob Kim, MD (Zarathu) for advice on statistical analysis.

## Author contributions

**Conceptualization:** Seunghwa Lee, Bum Sung Kim.

**Data curation:** Seunghwa Lee, Kyoung Ree Lim.

**Methodology:** Seunghwa Lee, Kwang Jin Chun, Bum Sung Kim.

**Writing – original draft:** Seunghwa Lee, Kwang Jin Chun.

**Writing – review & editing:** Kyoung Ree Lim, Bum Sung Kim.
